# Editorial: Advances in biomaterials and (Bio)fabrication for medical implants

**DOI:** 10.3389/fbioe.2026.2015618

**Published:** 2026-10-15

**Authors:** Xiaoyu Du, Xiao Kuang, Gurdial Blugan, Stephen J. Ferguson

**Affiliations:** 1 Institute for Biomechanics, ETH Zurich, Zurich, Switzerland; 2 Department of Mechanical Engineering, University of Wisconsin-Madison, Madison, WI, United States; 3 Laboratory for High Performance Ceramics, Empa, Swiss Federal Laboratories for Materials Science and Technology, Dubendorf, Switzerland

**Keywords:** biofabirication, biomaterials, implant, interface, medical device

Medical implants are expected to perform increasingly complex functions. Beyond providing mechanical support or replacing damaged tissues, modern implants must interact appropriately with the surrounding biological environment, resist infection, promote tissue integration, and maintain their function over long periods. At the same time, advances in additive manufacturing, surface engineering, hydrogels, nanomaterials, and computational modeling are providing new possibilities to address these requirements.

This Research Topic, *Advances in Biomaterials and (Bio)fabrication for Medical Implants*, brings together ten studies covering orthopedic, dental, cardiovascular, and veterinary applications. Although the investigated materials and clinical indications differ, the contributions share a common objective: improving the interaction between implant design, mechanical function, and the biological environment.

Mechanical reliability remains the foundation of implant design. Losey and Maritato characterized the screw retention and bending behavior of polyaxial combination-hole locking plates, providing mechanical data relevant to fracture fixation. Sim et al. approached implant mechanics from a computational perspective, investigating verification, validation, and uncertainty quantification of finite element models of pedicle screw assemblies. Their work demonstrates how assumptions at implant interfaces can substantially influence predicted mechanical behavior and highlights the importance of experimental validation when computational models are used in medical-device development.

Several contributions focus on the interface between metallic implants and surrounding tissue. Hertzberg-Boelch et al. investigated calcium hydroxide-coated titanium implants and reported increased bone–implant contact *in vivo*, together with potential antibacterial effects. Chen et al. explored nano-thin amorphous tantalum coatings on titanium implants for acetabular bone-defect reconstruction, combining preclinical evaluation with initial clinical observations. These studies show that the surface of an established structural material such as titanium can be modified to influence biological responses without fundamentally changing the underlying implant architecture.

This interface is also where one of the most persistent complications of medical implants occurs: infection. Liu et al. reviewed mechanisms of bacterial biofilm resistance on orthopedic implants and current strategies for anti-biofilm coatings. Brümmer et al., using a multispecies peri-implantitis biofilm model, demonstrated the potential of locally applied antibiotics for reducing bacterial biofilms across different implant materials. Together, these studies highlight both the importance of preventing implant-associated biofilm formation and the potential of targeted local antimicrobial strategies for managing established infections.

Other contributions illustrate how soft and composite biomaterials can introduce functions that are difficult to achieve with conventional metallic implants. Meng et al. developed an injectable, *in-situ* curable hydrogel for hemostasis within the medullary cavity, combining injectability with rapid curing and mechanical stability. Rezvova et al. investigated carbon-nanotube-reinforced polyvinyl alcohol cryogels for cardiovascular applications, demonstrating how nanoscale reinforcement can be used to tune the mechanical behavior of highly hydrated materials.

Biofabrication further enables control over both material architecture and biological function. Chang et al. used coaxial electrospinning to produce poly(ε-caprolactone)/gelatin core–shell scaffolds for localized delivery of metronidazole and dexamethasone, combining antibiofilm and anti-inflammatory functions within a single construct. Sallustio et al. developed a load-bearing vascular graft combining an elastin-like recombinamer hydrogel with a silk fibroin textile structure, illustrating how different biomaterial classes can be integrated to reproduce demanding mechanical and biological functions.

Taken together, the contributions to this Research Topic highlight an important direction in implant research: mechanics, materials, manufacturing, and biology cannot be optimized independently. An implant may possess excellent mechanical properties but fail because of poor tissue integration or infection. Conversely, a highly bioactive material has limited clinical value if it cannot withstand physiological loading, be manufactured reproducibly, or be incorporated into a practical implant design.

This is particularly relevant as implant development moves toward increasingly multifunctional systems. Surface coatings can modify the response of established metallic implants; hydrogels and composite materials can introduce tissue-like mechanical behavior or local therapeutic functions; advanced fabrication methods can control architecture across multiple length scales; and computational models can help evaluate designs before extensive experimental testing. The challenge is increasingly not whether each of these technologies can work individually, but how they can be integrated into robust and clinically translatable medical devices.

The studies collected in this Research Topic provide examples of this integration across different implant applications and stages of development. Continued collaboration between materials scientists, engineers, biologists, clinicians, and manufacturers will be essential to translate these advances into implants that are not only mechanically reliable, but also better adapted to the biological environments in which they must function.

We thank all authors for their contributions to this Research Topic, as well as the reviewers whose comments and expertise supported the publication of these studies.

